# A retrospective, multicentric, nationwide analysis of the impact of splenectomy on survival of pancreatic cancer patients

**DOI:** 10.1007/s00423-024-03570-y

**Published:** 2024-12-22

**Authors:** Maximilian Kießler, Carsten Jäger, Carmen Mota Reyes, Ilaria Pergolini, Stephan Schorn, Rüdiger Göß, Okan Safak, Marc E. Martignoni, Alexander R. Novotny, Waldemar Uhl, Jens Werner, Michael Ghadimi, Werner Hartwig, Reinhard Ruppert, Tobias Keck, Christiane J. Bruns, Karl-Jürgen Oldhafer, Andreas Schnitzbauer, Christoph-Thomas Germer, Florian Sommer, Sören Torge Mees, Maximilian Brunner, Jörg Köninger, Tim R. Glowka, Jörg C. Kalff, Christoph Reißfelder, Detlef K. Bartsch, Thomas Kraus, Winfried Padberg, Pompiliu Piso, Bernhard J. Lammers, Hagen Rudolph, Christian Moench, Stefan Farkas, Helmut Friess, Güralp O. Ceyhan, Ihsan Ekin Demir

**Affiliations:** 1 Department of Surgery, TUM Universitätsklinikum Klinikum Rechts der Isar Technische Universität München, Ismaninger Str. 22, 81675 Munich, Germany; 2 German Cancer Consortium (DKTK), Partner Site, Munich, Germany; 3 CRC 1321 Modelling and Targeting Pancreatic Cancer, Munich, Germany; 4 Else Kröner Clinician Scientist Professor for Translational Pancreatic Surgery, Munich, Germany; 5 Department of General Surgery, HPB-Unit, School of Medicine, Acibadem Mehmet Ali Aydinlar University, Istanbul, Turkey; 6 Department of General and Visceral Surgery, St. Josef Hospital, Ruhr-University Bochum, Bochum, Germany; 7 Department of General, Visceral, and Transplant Surgery, LMU University Hospital of Munich, Munich, Germany; 8 Department of General-, Visceral-, and Pediatric Surgery, University Medical Center Goettingen, Goettingen, Germany; 9 Department of General and Visceral Surgery, Evangelisches Krankenhaus Düsseldorf, Düsseldorf, Germany; 10 Department of General and Visceral Surgery, Endocrine Surgery, and Coloproctology, Municipal Hospital of Munich-Neuperlach, Munich, Germany; 11 Department of Surgery, University Hospital of Schleswig Holstein (UKSH), Lübeck, Germany; 12 Department of General, Visceral, Cancer and Transplantation Surgery, University of Cologne, Cologne, Germany; 13 Department Für Chirurgie, Klinik Für Leber-, Gallenwegs- Und Pankreaschirurgie, Asklepios Klinik Barmbek, Hamburg, Germany; 14 Clinic for General and Visceral Surgery, University Hospital Frankfurt, Goethe-University, Frankfurt/Main, Germany; 15 Department of General, Visceral, Transplantation, Vascular and Pediatric Surgery, Center of Operative Medicine (ZOM), University Hospital of Wuerzburg, Würzburg, Germany; 16 Department of General, Visceral and Transplantation Surgery, University Hospital Augsburg, Augsburg, Germany; 17 Department of General and Visceral Surgery, Dresden-Friedrichstadt General Hospital, Dresden, Germany; 18 Department of Surgery, University Hospital Erlangen, Erlangen, Germany; 19 Department of General, Visceral, Thoracic, and Transplantation Surgery, Klinikum Stuttgart, Stuttgart, Germany; 20 Department of Surgery, University Hospital of Bonn, Bonn, Germany; 21 Department of Visceral-, Thoracic and Vascular Surgery, University Hospital Carl Gustav Carus, Technical University Dresden, Germany; Department of Surgery, University Medicine Mannheim, Medical Faculty Mannheim, University of Heidelberg, Mannheim, Germany; 22 Department of Visceral, Thoracic and Vascular Surgery, Philipps-University Marburg, Marburg, Germany; 23 Hospital Nordwest GmbH, Frankfurt, Germany; 24 Department of General & Thoracic Surgery, University Hospital of Giessen, Giessen, Germany; 25 Department of General and Visceral Surgery, Hospital Barmherzige Brüder, Regensburg, Germany; 26 Department of General Surgery, Endocrine Surgery, Abdominal Surgery, Thorax Surgery, Vascular Surgery, Colorectal and Hernia Surgery, Lukaskrankenhaus GmbH, Neuss, Germany; 27 Department of General Und Visceral Surgery, Klinikum Chemnitz gGmbH, Chemnitz, Germany; 28 Department of General, Visceral, and Transplantation Surgery, Westpfalz-Klinikun GmbH, Kaiserslautern, Germany; 29 Department of General Und Visceral Surgery, St. Josefs-Hospital, Wiesbaden, Germany

**Keywords:** Pancreatic cancer, Splenectomy, Total pancreatectomy, Distal pancreatectomy

## Abstract

**Objective:**

Splenectomy is regularly performed in total and distal pancreatectomy due to technical reasons, lymph node dissection and radicality of the operation. However, the spleen serves as an important organ for competent immune function, and its removal is associated with an increased incidence of cancer and a worse outcome in some cancer entities (Haematologica 99:392–398, 2014; Dis Colon Rectum 51:213–217, 2008; Dis Esophagus 21:334–339, 2008). The impact of splenectomy in pancreatic cancer is not fully resolved (J Am Coll Surg 188:516–521, 1999; J Surg Oncol 119:784–793, 2019).

**Methods:**

We therefore compared the outcome of 193 pancreatic cancer patients who underwent total or distal pancreatectomy with (Sp) or without splenectomy (NoSp) between 2015 and 2021 using the StuDoQ|Pancreas registry of the German Society for General and Visceral Surgery. In addition, we integrated our data into the existing literature in a meta-analysis of studies on splenectomy in pancreatic cancer patients.

**Results:**

There was no difference between the Sp and NoSp groups regarding histopathological parameters, number of examined or affected lymph nodes, residual tumor status, or postoperative morbidity and mortality. We observed a significantly prolonged survival in pancreatic cancer patients who underwent total pancreatectomy, when a spleen-preserving operation was performed (median survival: 9.6 vs. 17.3 months, *p* = 0.03). In this group, splenectomy was identified as an independent risk factor for shorter overall survival [HR (95%CI): 2.38 (1.03 – 6.8)]. In a meta-analysis of the existing literature in combination with our data, we confirmed splenectomy as a risk factor for a shorter overall survival in pancreatic cancer patients undergoing total pancreatectomy, distal pancreatectomy, or pancreatic head resection [HR (95%CI): 1.53 (1.11 – 1.95)].

**Conclusion:**

Here, we report on a strong correlations between removal of the spleen and the survival of pancreatic cancer patients undergoing total pancreatectomy. This should encourage pancreatic surgeons to critically assess the role of splenectomy in total pancreatectomy and give rise to further investigations.

**Supplementary Information:**

The online version contains supplementary material available at 10.1007/s00423-024-03570-y.

## Introduction

Multiple studies have reported on the outcome of cancer patients when a splenectomy was performed as part of tumor resection. In colorectal cancer, avoidance of splenectomy was associated with superior patient survival [1]. For esophageal cancer, Pultrum et al. observed a significant difference in the 2-year survival rate, favoring the no-splenectomy group [2]. In 1999, Schwarz et al. described a survival benefit of pancreatic cancer patients when a spleen preserving pancreatic resection was performed [3]. Still, splenectomy is often performed as part of total or distal pancreatic resection. The reasons include technical difficulties of spleen preservation, radicality of the operation, tumors adjacent to splenic hilus, and completeness of lymph node removal. Several studies proved that preservation of the spleen is feasible and safe in pancreatic cancer patients [4–7]. Direct infiltration of the spleen can be assessed reliably by preoperative CT scan [8]. Also, the number of lymph nodes in the splenic hilus is rather small, and they are rarely affected by pancreatic cancer [8–10]. In addition, recent data suggest an impaired anti-tumor immune response after splenectomy in murine pancreatic cancer models [11, 12]. We therefore analyzed the impact of splenectomy on the long-term outcome of pancreatic cancer patients undergoing total or distal pancreatectomy.

## Material and methods

### Retrospective registry study and survival analysis

We retrospectively analyzed data from the *Studies, Documentation, and Quality Center* (*Studien-, Dokumentations- und Qualitätszentrum, StuDoQ*) of the *German Society for General and Visceral Surgery* (*DGAV*) from 2015—2021. Patients with histologically confirmed pancreatic ductal adenocarcinoma, who underwent either total or distal pancreatic resection with curative intention, were included in the study. This study was approved by the ethics committee of the Technical University Munich (2022–408-S-NP).

Group comparison (splenectomy yes vs. no) was done by chi-square or student’s-t test, when applicable. Kaplan-Meier curves were used to illustrate patient survival, with log rank test for statistical differences. A Cox proportional hazards regression model was used for multivariate analysis of patient survival regarding splenectomy and histopathological features.

### Meta-analysis

A systematic literature search was performed in relevant databases (Google Scholar, PubMed, Cochrane Library) for the search term [("pancreatic cancer" OR "pancreatic ductal adenocarcinoma" OR "pancreatic malignancy") AND ("Splenectomy") AND ("Distal pancreatectomy" OR "total pancreatectomy" OR "left pancreatectomy") AND ("Outcome" OR "Outcomes")]. A total of 4.539 Studies (PubMed 74, Google Scholar 4.460, Cochrane Library 5) were identified. After title and abstract screening, only three studies could be identified addressing this topic, from which two included sufficient outcome data to be implemented in a meta-analysis. Hazard ratio and standard error was approximated by given *p*-values, number of included patients, and noted observations. A random effects model was used to calculate a combined hazard ratio (HR). All statistical analysis was performed using the software R and R Studio.

## Results

### Patient cohort

We included a total of 193 patients with histologically confirmed pancreatic ductal adenocarcinoma who underwent either distal or total pancreatectomy in our analysis (for all details see Table 1). The mean age of the patient cohort was 66.2 years, 59% of the patients were male. There was no significant difference in the distribution of patients by sex or gender across the two groups. Clinically, there was no difference in new-onset diabetes or preoperative CA19-9 levels.

**Table 1:** Patient cohort characteristic and comparison of histopathological parameters between the two patient cohorts with and without splenectomy. Chi-square or student’s-t test was used for the calculation of *p* values

	No Splenectomy	Splenectomy	*P* value
Number of patients	107	86	
Sex of the Patients (male/female)	59/48	49/37	0.91
Mean age of Patients (years)	66.4	65.8	0.71
Newly diagnosed diabetes (%)	10.3	7.0	0.58
Preoperative CA19-9 levels	223.9	430.1	0.10
Number of total pancreatectomies	51	39	
Number of distal pancreatectomies	56	47	
pT			0.54
pT0	3	0	
pT1 (n)	13	10	
pT2 (n)	35	28	
pT3 (n)	45	42	
pT4 (n)	10	6	
pTx	1	0	
pN			0.33
pN0 (n)	43	31	
pN1 (n)	47	34	
pN2 (n)	17	21	
R			0.12
R0 (n)	74	60	
R1 (n)	24	25	
R2 (n)	2	0	
Rx (n)	7	1	
G			0.19
G1 (n)	1	2	
G2 (n)	43	33	
G3 (n)	30	34	
Gx (n)	33	17	
L			0.67
L0 (n)	55	45	
L1 (n)	51	41	
Lx (n)	1	0	
Pn			0.14
Pn0 (n)	27	13	
Pn1 (n)	79	73	
Pnx (n)	1	0	
V			0.60
V0 (n)	72	34	
V1 (n)	61	25	
Vx (n)	1	0	
Number of examined lymph nodes (median, range)	28.1 (2 – 115)	27.0(1 – 63)	0.66
Number of affected lymph nodes (median, range)	2.3 (0 – 32)	3.0 (0 – 37)	0.33
Percent affected lymph nodes (median, range)	17% (0 – 100)	14% (0 – 70)	0.67
Mean operating time overall (minutes)	337.9	339.0	0.96
Mean operating time distal pancreatectomy (minutes)	276.0	252.9	0.33
Mean operating time total pancreatectomy (minutes)	405.8	442.8	0.16
Mean intraoperative blood transfusion (number)	0.64	0.56	0.74
Postoperative bleeding (%)	13.1	10.5	0.74

Splenectomy was performed in 45.6% (47/103) of the patients undergoing distal pancreatectomy and in 43.4% (39/90) of the patients undergoing total pancreatectomy. For all distal pancreatectomies the spleen preservation was performed by Warshaw procedure. Overall, the two groups exhibited no significant difference in operation time (Sp: 339.0 min, noSp: 337.9 min, *p* = 0.96). The mean operating time for distal pancreatectomy was 23.2 min longer in the no splenectomy group (sp: 252.9 min, noSp: 276.0 min, *p* = 0.33). In the case of total pancreatectomy, the mean operating time was found to differ by 37.0 min, with splenectomy being the slower operation, although this difference did not reach statistical significance (Sp: 442.8 min, noSp: 405.8 min, *p* = 0.16). Furthermore, no significant differences were observed in intraoperative blood transfusions (Sp: 0.64, noSp: 0.56, *p* = 0.74), or the incidence of postoperative bleeding, defined as the requirement for more than three blood transfusions during the postoperative course (Sp: 10.5, noSp: 13.1, *p* = 0.74).

There was no difference in tumor size (pT), involvement of lymph nodes (pN), grading (G), or resection status (R) between the two groups. The mean number of lymph nodes examined in the two groups were also comparable [Sp: 27 (range 1—63), noSp: 28 (range 2 – 115), *p* = 0.66]. Further, there was no significant difference in the number of affected lymph nodes (Sp: 3.0, noSp: 2.3, *p* = 0.33) or the percentage of affected to total number of examined lymph nodes (Sp; 14.0%, noSp: 17.3%, *p* = 0.67). Additionally, the postoperative morbidity and mortality based on the Clavien Dindo Classification in the Sp and noSP group did not differ between the groups (supplementary Fig. 2).

### Splenectomy is associated with a shorter overall survival

**Fig. 1:**
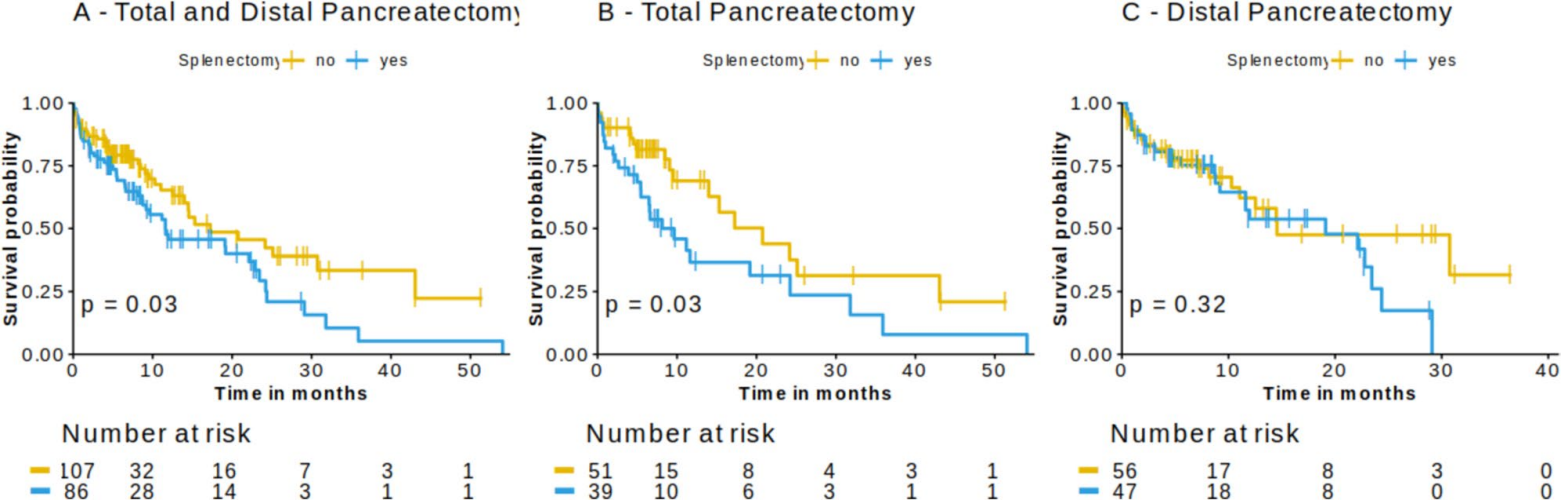
Preservation of the spleen is associated with a prolonged overall survival of pancreatic cancer patients who undergo total pancreatectomy. We observed a significantly longer overall survival in the combined and in the total pancreatectomy group. There was no significant survival difference in the distal pancreatectomy group. Kaplan-Meier curves illustrate patient survival. Log rank was used to calculate p-values

**Fig. 2:**
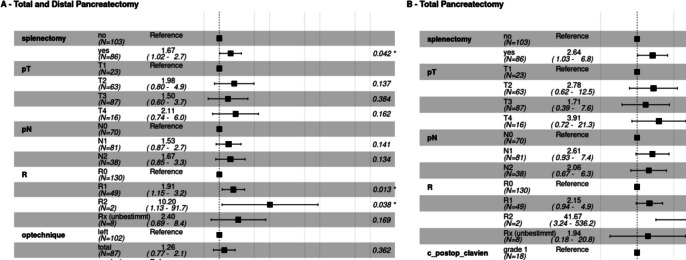
Multivariate analysis. Splenectomy as independent risk factor for a shorter overall survival of pancreatic cancer patients in the combined cohort (**A**) and the total pancreatectomy cohort alone (**B**). A Cox proportional hazards regression model was used for multivariate analysis. Four patients were excluded with pT0 or pTx as pT status due to small group size

Median survival of patients in the combined cohort of distal and total pancreatectomy was 11.6 months in the splenectomy group (*n* = 86), compared to 17.3 months in the control group (*n* = 107) with spleen-preserving operation (*p* = 0.03) (Fig. 1A). Here, splenectomy was associated with reduced patient survival (Hazard Ratio 1.7, 95%CI 1.05–2.2, *p* = 0.03) independent of pT, pN, R status (Fig. 2A). In the subgroup analysis of total and distal pancreatectomy, only total pancreatectomy showed a significant association with patient survival (*p* = 0.03) with a median survival of 9.6 vs. 20.8 months in the splenectomy vs. no splenectomy group, respectively (Fig. 1B). Further, splenectomy in patients who underwent total pancreatectomy was identified to be a highly prognostic marker for a shorter overall survival, independent of pT-, pN, R status, postoperative morbidity and mortality and center volume, in a multivariate analysis [HR (95%CI): 2.64 (1.03 – 6.8)] (Fig. 2C).

**Fig. 3:**
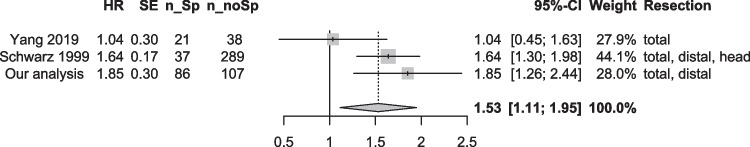
Meta-analysis of studies on long-term outcome after pancreatic resection with or without splenectomy. Random effects model shows an increased risk for shorter overall survival if splenectomy is performed. HR = Hazard Ratio, SE = Standard Error, n_Sp = number of operations with splenectomy, n_noSp = number of spleen-preserving operations

To fit our data into the existing literature, we conducted a meta-analysis of studies with data on long-term outcome after resected pancreatic cancer with information on splenectomy. In a systematic literature search, we identified two matching studies with sufficient data. Yang et al. did not observe a significant difference between the splenectomy and no splenectomy group in their retrospective analysis of patients undergoing total pancreatectomy for resection of pancreatic cancer [4]. In contrast, Schwarz et al. performed a retrospective study on patients undergoing resection of the pancreatic head, tail, or total resection and found that, independent of the performed operation, there was a significant survival difference between the two groups, in favor of patients without splenectomy [3]. We fitted the available data from the existing studies, including our own, into a meta-analysis. Also here, splenectomy was evident as risk factor for a shorter overall survival [HR (95%CI): 1.53 (1.11 – 1.95)] in a random effects model (Fig. 3).

## Discussion

Resection of the tumor is the only potentially curative treatment of pancreatic cancer. In the context of left or total pancreatectomy removal of the spleen is often performed due to the anatomical proximity of the spleen to the pancreatic tail and affection of the splenic vessels by the tumor. Spleen preservation can either be performed through sparing of the splenic artery and vein or by vessel resection. The preservation of the splenic vessels can be surgically challenging, especially in malignant pancreatic disease, where tumor free resection margins are a pivotal factor for patient outcome. In 1988 Warshaw originally described a pancreatic resection technique with preservation of the spleen but resection of the splenic vessels, based on the preservation of the short gastric- and the left gastroepiploic vessels taking advantage of splenic collateral blood flow [5]. Today, multiple studies have proven the safety and effectiveness of this procedure in open and laparoscopic surgery with no differences in early postoperative outcome [6, 13–16]. Our findings align with this conclusion, as we could not identify any disparities in postoperative morbidity and mortality (see supplementary Fig. 2). Additionally, no significant differences were observed in terms of operative time or perioperative blood loss in the patient cohort under investigation. In total pancreatectomy, where spleen preservation is most challenging, we observed a shorter operation time compared to simultaneous splenectomy, although this difference did not reach statistical significance. This is probably due to the fact that the Warshaw technique was performed in all cases, eliminating the need for time-consuming exposure of the splenic vessels.

Besides technical reasons, a radical lymph node dissection, including the lymph nodes in the hilus of the spleen, is a reason for splenectomy in pancreatic cancer surgery, especially because nodal involvement is directly correlated with patient outcome. In our study we did not observe any difference between in the number of examined and affected lymph nodes between the splenectomy and no splenectomy group. This is in line with a retrospective analysis of lymph node involvement in patients who underwent total splenopancreatectomy by Collard et al., who found a low median number of only two lymph nodes in the splenic hilus, with 40% of the patients with no lymph nodes detectable at all. None of the hilus lymph nodes were positive for metastasis in this study [8]. Navez et al. described only one case of positive lymph node metastasis in a group of 85 patients with pancreatic adenocarcinoma of the body or the tail, and Kim et al. reported four positive hilar lymph nodes in a cohort of 97 cases of pancreatic cancer [9, 10].

Further, direct infiltration of the spleen by pancreatic tumors of the tail presents a reason for splenic removal. It has been shown that this can be reliably predicted in the preoperative CT [8]. Therefore, cases for potential spleen preservation should be preoperatively assessed for direct splenic infiltration. In our patient cohort, we did not observe any difference in the residual tumor/margin status between the splenectomy and no splenectomy group.

In a large cohort study of cancer-free veterans by Kristinson et al., splenectomy was associated with an increase in cancer incidence and risk of cancer death [17]. This observation was confirmed by Sun et al. in their population-based cohort study of patients undergoing splenectomy for traumatic and nontraumatic indications [18]. In both groups, splenectomy was associated with a higher risk for overall cancer development [18]. When splenectomy was a part of tumor resection of patients with colorectal or esophageal cancer, it was associated with poorer patient prognosis [1, 2]. In pancreatic cancer, Schwarz et al. reported a significantly prolonged survival of patients who underwent a spleen-preserving operation for pancreatic cancer with curative intention. These results are in line with our data; however, Schwarz et al. included distal and total pancreatectomies, and additionally pancreatic head resections (pancreatoduodenectomy). They reported a median survival of 12.2 versus 17.8 months in the splenectomy (*n* = 37) vs. the spleen-preserving group (*n* = 289), respectively [3]. In their multivariate analysis, splenectomy was found to be an independent factor for a shorter survival. Interestingly, the survival benefit was independent of the performed operation [3]. In our data, the survival benefit of patients without splenectomy was prominent in the total pancreatectomy group, while we did observe little to no effect in the distal pancreatectomy group. The same observation was reported in a Dutch outcome analysis of patients with pancreatic cancer who underwent distal pancreatectomy. In line with our data, splenectomy did not show a significant association with patient survival in this subgroup [19]. However, the proportion of spleen-preserving compared to spleen-resecting surgery in this study was rather small (17 vs. 124) and the study may therefore lack the statistical power to detect survival differences [19]. In contrast to our data, the retrospective analysis of Yang et al. did not detect a difference in long-term survival of pancreatic cancer patients undergoing total pancreatectomy between the spleen preservation (Warshaw technique) and splenectomy group (*n* = 38 vs. 21, *p* = 0.905) [4]. We analyzed the above-mentioned studies from Schwarz et al. [3] and Yang et al. [4] in a meta-analysis (Rooij et al. [19] did not include *p* values). Here, splenectomy was identified as an independent risk factor for an inferior long-term survival. However, our study is certainly limited by its retrospective nature. It is crucial to acknowledge that a significant constraint of this study is the use of overall patient survival data. While overall survival and progression-free survival are closely correlated for pancreatic cancer patients, given the unfavorable prognosis and high recurrence rate, this introduces another potential source of bias that limits the generalisability of our conclusions. Additionally, information on tumor location and indication for total pancreatectomy (tumor location, size, high-risk pancreaticojejunostomy, or no residual functioning pancreas), indication for the chosen technique (spleen preservation/resection), and perioperative treatment regime (neoadjuvant/adjuvant treatment) was not included in the StuDoQ|Pancreas registry. Another data that was not sufficiently reported in the database was the information on pre- and postoperative oncologic treatment. This remains a major limitation of the study, as this is of crucial importance to the tumor recurrence and with that to overall survival. Due to the limitations mentioned above, we cannot make a general recommendation for spleen preservation in ductal pancreatic cancer. Prospective, randomized trials with recurrence-free survival data are needed to provide clear guidelines.

Our systematic review shows that the impact of spleen removal on pancreatic cancer outcomes is poorly studied and the underlying mechanisms remain unknown. In the human organism, hematologic changes can be observed after splenectomy [20]. However, it is not known how this affects the course of malignant disease, such as pancreatic cancer. One pioneering study by Hwang et al. observed a significant increase in tumor growth after removal of the spleen in their murine pancreatic cancer model [11]. In the splenectomy group, they additionally observed a lower ratio of effector T cells (CD8 + /CD4 +) to immunosuppressive regulatory T cells (FOXP3 +), suggesting an impaired anti-tumor immune response [11]. It may be that this impaired immune system leads to a less effective anti-tumor immune response, especially when severe diabetes co-exists e.g. after total pancreatectomy [21]. However, further evidence is needed to understand and potentially circumvent these changes induced by splenectomy.

## Conclusion

In conclusion, spleen preservation is associated with a prolonged survival of pancreatic cancer patients undergoing total pancreatectomy independent of other established risk factors. Our data is in line with a few previous reports on a survival benefit, when a spleen-preserving surgery is performed.

However, as a result of the above-mentioned limitations, no reliable causality between splenectomy and a survival benefit of patients after pancreatic surgery can be established based on our results. Nevertheless, the strong correlations found should encourage pancreatic surgeons to critically assess the role of splenectomy in total pancreatectomy and give rise to further investigations.

## Supplementary Information

Below is the link to the electronic supplementary material.

Supplementary file1 (PDF 101 KB)

## Data Availability

No datasets were generated or analysed during the current study.
