# Supplementary material for: A retrospective, multicentric, nationwide analysis of the impact of splenectomy on survival of pancreatic cancer patients
Source: Langenbecks Arch Surg. 2024 Dec 22;410(1):14. doi: 10.1007/s00423-024-03570-y (PMC11663811; doi:10.1007/s00423-024-03570-y)
Supplement: Supplementary file 1 — Supplementary file1 (PDF 101 KB) [file 423_2024_3570_MOESM1_ESM.pdf]

Supplementary Material

Supplementary Figure 1

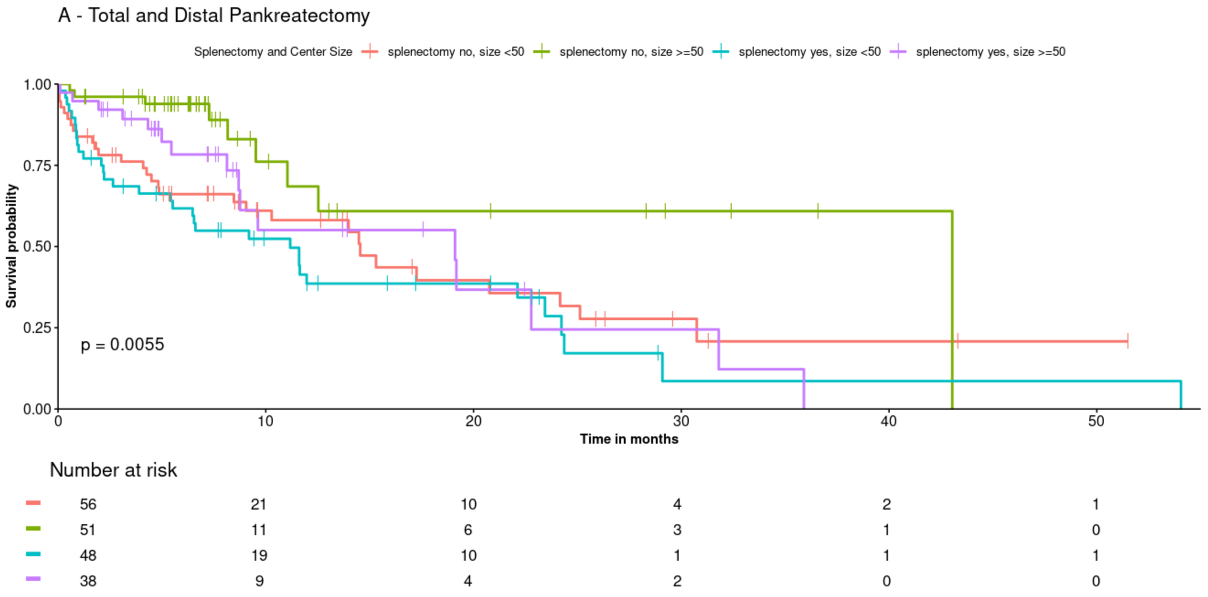

Survival analysis of splenectomy in combination with center size. The Kaplan-Meier curves illustrate, that patients have the best prognosis in high volume centers (>=50 cases/year). However, for both center volumes, spleen preservation remains an independent factor for a better outcome of the patient.

Supplementary Figure 2

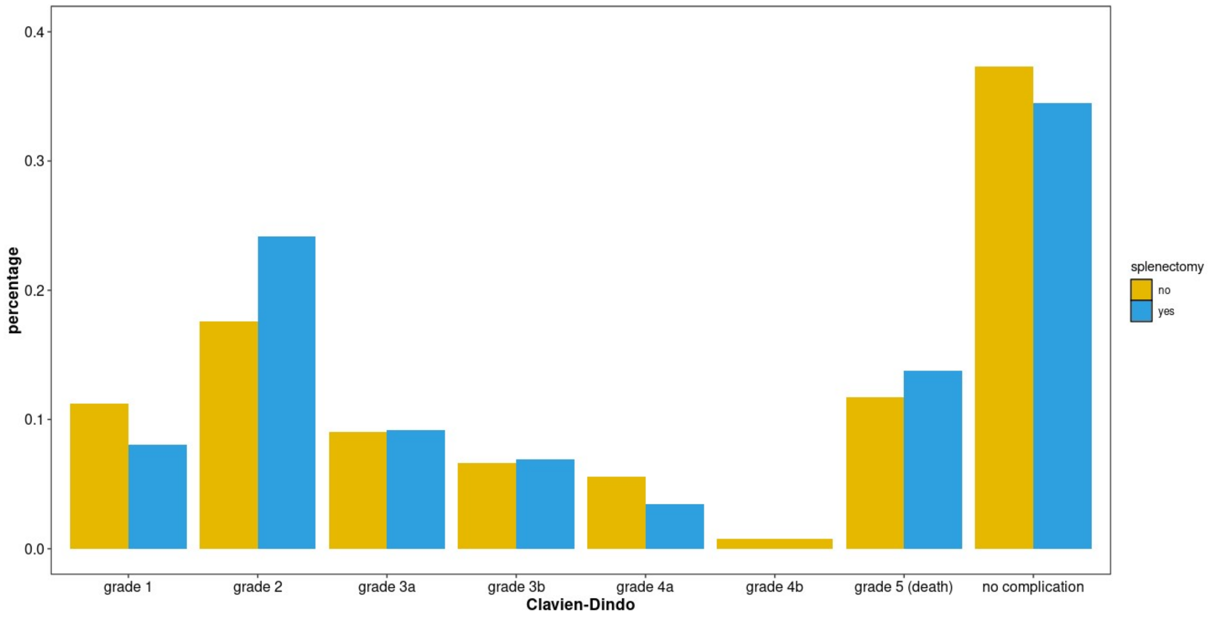

Comparison of postoperative morbidity and mortality based on Clavien Dindo Classification in the splenectomy and spleen-preserving group. No significant differences could be found between the two groups.
